# Comprehensive Analysis of Regulatory Networks of m6A Regulators and Reveals Prognosis Biomarkers in Sarcoma

**DOI:** 10.3389/fonc.2022.911596

**Published:** 2022-07-01

**Authors:** Boran Pang, Dinghao Luo, Bojun Cao, Wen Wu, Lei Wang, Yongqiang Hao

**Affiliations:** ^1^Shanghai Key Laboratory of Orthopaedic Implants, Department of Orthopaedic surgery, Shanghai Ninth People’s Hospital, Shanghai Jiao Tong University School of Medicine, Shanghai, China; ^2^Clinical and Translational Research Center for 3D Printing Technology, Shanghai Ninth People’s Hospital, Shanghai Jiao Tong University School of Medicine, Shanghai, China

**Keywords:** m6A, regulatory network, sarcoma, prognosis, immune infiltration

## Abstract

Sarcomas are rare malignant tumors that may arise from anywhere of the body, such as bone, adipose, muscle and vascular. However, the conventional pathogenesis of sarcomas has not been found. Therefore, there is an urgent need to identify novel therapeutic strategies and improve prognosis effects for sarcomas. Methylation of N6 adenosine (m6A) regulation is a novel proposed regulatory pattern that works in post-transcription level, which was also the most widely distributed methylation modification in eukaryotic mRNA. Growing evidences have demonstrated that m6A modification played an indispensable role in tumorigenesis. Here, we integrated multi-omics data including genetic alterations, gene expression and epigenomics regulation to systematically analysis the regulatory atlas of 21 m6A regulators in sarcoma. Firstly, we investigated the genetic alterations of m6A regulators and found that ~44% TCGA sarcoma patients have genetic mutations. We also investigated the basic annotation of 21 regulators, such as expression correlation and PPI interactions. Then we identified the upstream and downstream regulatory networks of between transcription factors (TFs)/non-coding RNAs and m6A regulators in sarcoma based on motif analysis and gene expression. These results implied that m6A regulator mediated regulatory axes could be used as prognostic biomarkers in sarcoma. Knockdown experiment results revealed that m6A regulators, YTHDF2 and HNRNPA2B1 participated in the cancer cell invasion and metastasis. Moreover, we also found that the expression levels of m6A regulators were related to immune cell infiltration of sarcoma patients.

## Introduction

Sarcomas are a large category of cancers that arise from mesenchymal cells, which can origin in almost any tissue, including bone, adipose, or muscle (1). For example, bone sarcomas are the most frequent primary solid malignancy of mesenchymal origin characterized by malignant spindle stromal cells that produce bone-like tissue (2). Sarcomas are morphologically heterogeneous, accurate diagnosis of which require an integrated strategy to consider and assess the clinical, molecular, and histologic characteristics of the malignancy (3). In addition, the malignant degree of sarcomas is high, most patients develop metastasis within one year, and the prognosis is rather poor (4). Understanding the molecular mechanisms of sarcoma development is urgent.

Methylation of N6 adenosine (m6A) is the most abundant internal chemical modification in eukaryotic mRNA (5). m6A modification was firstly discovered in 1970s and the detailed functional studies of m6A modification began 2012. Now, m6A modification becomes the most prevalent study of RNA modification that has received increasing attention. A large number of studies have suggested that aberrant m6A modification is the key to tumorigenesis and progression, such as breast cancer, lung cancer, acute myeloid leukemia and hepatocellular carcinoma (HCC) (6–8). The abundances and effects of m6A modification on RNAs are determined by the complex interactions between different types of regulators, including methyltransferases (‘writers’), RNA binding proteins (‘readers’), and demethylases (‘erasers’). Understanding these different m6A regulators could dramatically increase our knowledge about the role of RNA methylation in the regulation of gene expression and various biological processes (9, 10). Recently, lots of studies have demonstrated that m6A regulators were widely perturbed in various types of cancers (11, 12). For example, a component of the m6A methyltransferase complex, methyltransferase-like 3 (METTL3), was reported to be associated with translation machinery and promote the translation of oncogenes (*RGFR* and *TAZ*) in human lung cancer (13). The overexpression of METTL3 was also observed in HCC and was associated with poorer survival (14). The overexpression of METTL14 was shown to increase the abundance of m6A methylation on primary *miR-126*, which suppresses metastasis in HCC and breast cancer (15). Aberrant expression of FTO (m6A eraser) was suggested to be favorable for the survival of diverse cancer cells. And the overexpression of FTO could contribute to the proliferation and invasiveness of gastric cancer, squamous cell, and breast cancer cell lines (16–18). A family of m6A reader proteins IGF2BP1-3 was reported to have oncogenic potential, which was frequently expressed and amplified in cervical or liver cancers (19). All these findings provide strong evidence that m6A regulators play crucial roles in the development and progression of cancers. However, though recent discoveries of the functions and mechanisms of m6A have clarified a new perspective of gene regulation at the RNA level, we still lack a vast amount of knowledge about the functions of m6A regulators in the development and progression of sarcomas.

Therefore, in this study, we emphatically discussed and analyzed the important roles of m6A regulators in sarcomas from a global perspective based on integrative analyses. Firstly, we integrated multi-omics data to analyze the genetic alterations, gene expression and epi-genomics regulation of the of 21 m6A regulators in sarcoma. Secondly, we illustrated the potentials of m6A regulators in tumor immunology, paving the way for the therapeutic strategies of sarcomas based on RNA methylation. We also investigated the associations between the expression of m6A regulators and sarcoma patient survival and explored the clinical prognostic values of m6A regulators. Importantly, we constructed the regulatory networks for m6A regulators by integrating upstream and downstream regulatory information, including regulatory TFs and non-coding RNAs. And we performed knockdown experiments for YTHDF2 and HNRNPA2B1 to reveal the biological role in the cancer cell invasion and metastasis. Moreover, we also investigated the relationship between m6A regulators and immune cell infiltration in sarcoma patients. Our comprehensive analysis of m6A regulators would provide new insights into their function in the mechanism and development of sarcomas.

## Materials and Methods

### Gene Expression and Genetic Alterations of Sarcoma

The TCGA sarcoma gene expression data and matched clinical data were downloaded from XENA browser (https://xenabrowser.net/hub/). Based on the data processing pipeline to remove null data, sarcoma-related RNA-seq data containing 265 samples with clinical information were used for further study. TCGA sarcoma genetic alteration data were downloaded from cBioportal for Cancer Genomics. List of m6A regulators were downloaded from the previous study. All m6A regulators related gene expression data, clinical data and genetic alteration data were extracted from above data. The raw clinical data was provided in **Supplementary Table S1**.

### Annotation for m6A Regulators in Sarcoma

In this study, we performed multiple annotation analyses for m6A regulators, such as gene expression comparison, gene correlation and crosstalk network. Gene expression comparison was performed to compare regulator expression in control and tumor groups in TCGA cohorts *via* gglopt2. Pearson correlation analysis was performed on TCGA expression data *via* Corrplot. Gene crosstalks were downloaded from String database (https://www.string-db.org/).

### Survival Analysis

Hazards Ratio (HR) analysis of m6A regulators in sarcoma was analyzed from GEPIA2 database (http://gepia2.cancer-pku.cn/#survival) based on using Mantel–Cox test. For single gene survival analysis, patients were classified into high-Exp group and low-Exp groups based on mean expression. For multiple gene survival analysis, a risk score model was constructed. The risk score for each patient was computed by linear combination of the gene expression values weighted by the regression coefficient of univariate Cox regression analysis, which was defined as follows:


$$\text{RiskScore}=\sum_{\text{i}=1}^{\text{n}}{\text{r}}_{\text{i}}\text{Exp}\left(\text{i}\right)$$

Where, r_i_ is the Cox regression coefficient of gene i in gene set, n is the number of genes in gene set and Exp (i) is the expression value of gene i in corresponding patient. The mean risk score was used to classify patients into high-risk and low-risk groups. Kaplan-Meier survival curve was performed for high-risk and low-risk groups of patients *via* survival R package. The statistical significance was assessed by log-rank test with a threshold of P < 0.05.

### TF-m6A Regulator Regulatory Relationships

To identify upstream TFs of m6A regulators, we collected human enhancers from Fantom database and downloaded gene transcription start site (TSS) from UCSC database. For each regulator, we defined the Fantom enhancers located in 2000 bp~50000 bp far from of the TSS as the enhancers of the regulator. Promoters were defined as +/-2000 bp from regulator TSS. Find Individual Motif Occurrences (FIMO) software was used to scan TF motifs for each regulator’s enhancer and promoter at the threshold of P value <1e–4. If a TF located in the enhancer or promoter of the m6A regulator, we considered that this TF could regulate the m6A regulator, which forms a TF-m6A regulator interaction. All TF-m6A regulator interactions were merged to TF-m6A regulator regulatory network and were showed in Cytoscape 3.6.

### m6A Regulator-miRNA Regulatory Relationships

m6A regulators were demonstrated to play important roles in miRNA maturation and miRNA expression. Thus, matched TCGA miRNA expression profile was downloaded from XENA database and Pearson correlation was conducted to investigate the potential regulatory relationships between miRNAs and m6A regulators. m6A regulator-miRNA network was constructed by merging all positive correlated regulator-miRNA pairs with Pearson correlation coefficients (PCC) >0.4. Pathway enrichment was performed by miEAA.

### Immune Cell Infiltration of m6A Regulators in Sarcoma Patients

Infiltration estimation for all sarcoma patients were downloaded from TIMER2 database. The potential role of m6A regulators in cell infiltration was estimated by calculating the correlation between m6A regulator expression and infiltration estimation scores.

### Cell Culture

Ewing sarcoma cell lines (A673, SKNMC, TC32, TC71,EW8, TCC446 and EWS502) were used in this study and were cultured in 25 cm^2^ cell culture flask (Corning, NYC, USA) with Dulbecco’s Modified Eagle Medium (DMEM) (Invitrogen, Waltham, USA) containing 15% fetal bovine serum (Invitrogen, Waltham, USA) at 37°C, 5% CO_2_ environment. x-treme GENE siRNA (Invitrogen, Carlsbad, USA) were used for gene knockdown experiments for 24 h. siRNA sequences were provided in **Supplementary Table S2**.

### Quantitative Real-Time RT-PCR

Total RNA was extracted from cell lines using Trizol reagent (Invitrogen, Waltham, USA) according to manufacturer’s protocols. cDNA was synthesized by reverse transcription reagent kit (TAKARA, RR037A, Shiga, JAPAN). Gene expression was quantified by SYBR Green PCR Master Mix, and detected using Roche 480 systems. U6 or GAPDH was served as an internal control for miRNAs and mRNAs, respectively. 2^-ΔΔCt^ relative quantification method was used to show gene expression. Primers are listed in **Supplementary Table S3**.

### Western Blotting

Total protein was extracted from cell lines and lysed *via* RIPA buffer. Degenerated protein concentration was measured by bichinchoninic acid (BCA) Protein Assay Kit (Beyotime, Shanghai, China). In each experiment, 20 μg protein samples were separated in 10% or 15% SDS-PAGE gel and transferred onto nitrocellulose membrane. After 5% non-fat milk blocking, the blots were incubated with primary antibodies including YTHDF2 (1:1000 dilution, #71283, Cell signaling), HNRNPA2B1 (1:2000 dilution, #9304, Cell signaling), HNRNPC (1:1000 dilution, HPA051075, Sigma) and internal control α-Tubulin (1:2000 dilution, #3873, Cell signaling).

### Wound Healing Assay and Colony Formation Assays

For wound healing assay, cells were cultured in 6-well plates, and the cell monolayer was wounded by sterile 100-μL pipette tips when cells reached approximately 90% confluence. Cells were then rinsed three times with D-Hanks to wipe off the detached cells and were incubated in RPMI 1640 containing 5% FBS for 48 h. For colony formation assay, cells were also cultured in 6-well plates for 2–3 weeks. Resulting colonies were calculated following 1% crystal violet staining.

## Result

### Genetic Alterations Overview of m6A Regulator

**Figure 1:**
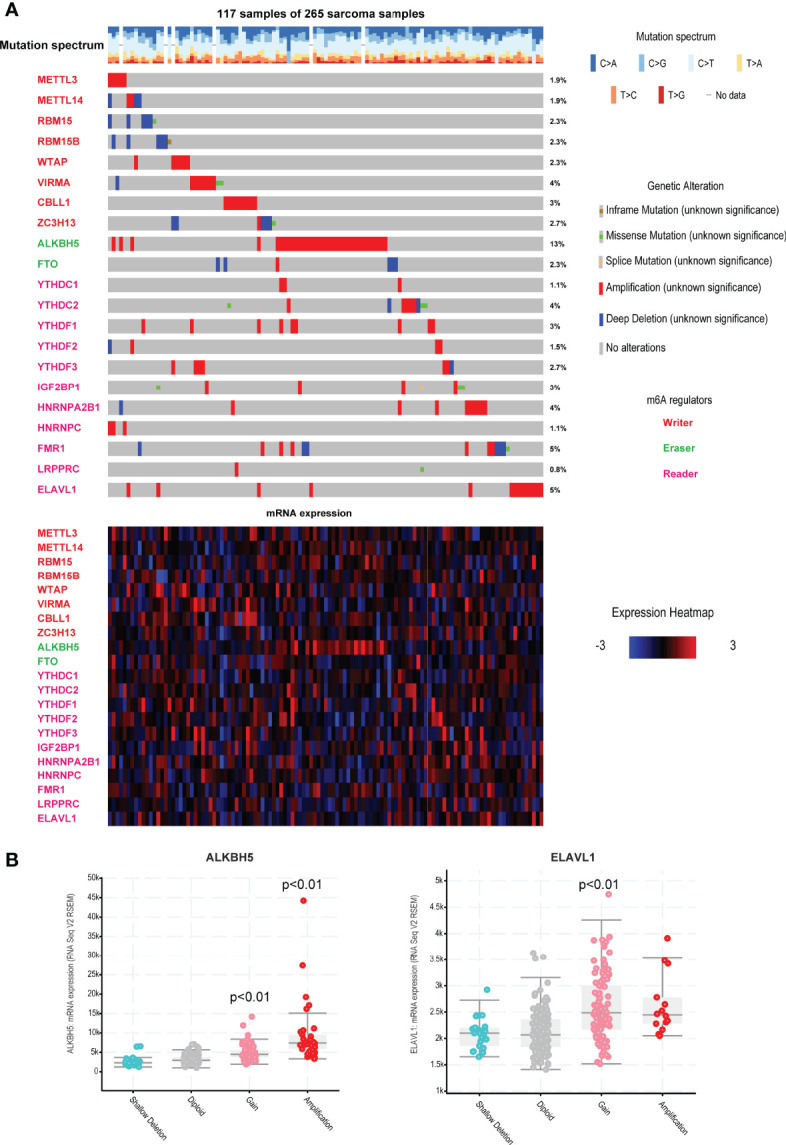
Landscape of genetic and expression variation of m6A regulators in TCGA sarcoma. **(A)** The mutation frequency of 21 m6A regulators in 265 patients with sarcoma in TCGA. Each column represented individual patients. The number on the right indicated the mutation frequency in each regulator. The lower heatmap represents m6A regulators’ expression in sarcoma. Three types of regulators were labeled in different colors. **(B)** The impacts of different genome alterations on gene expression of ALKBH5 and ELAVL1. *P*<0.01 represents the expression levels were significantly changed in alteration group vs. diploid group.

Here, we collected and analyzed 21 m6A regulators in this study, including 8 writers, 2 erasers and 11 readers. Firstly, we viewed the incidence of copy number variations and somatic mutations of the 21 regulators. Results showed that ~44% sarcoma patients (117 samples of 265 samples) carried mutations of m6A regulators (**Figure 1A**, top). Amplification is the most types of alterations, which could lead to the dysfunctional gene overexpression. It was found that ALKBH5 exhibited the highest mutation frequency of 13% and the patients with high amplification alteration also exhibited the high gene expression (**Figure 1A**, bottom and **Figure 1B**). ELAVL1 also showed the high mutation frequency of 5%. Alterations of ELAVL1 could also determine the ELAVL1 RNA expression. Some reader genes of LRPPRC, YTHDC1 and HNRNPC exhibited low mutation levels. These results demonstrated that genetic alterations could affect the expression of m6A regulators. Genetic alterations were also considered as the risk factor of multiple cancers, these results also implied that m6A regulators might be the driven factors of cancers.

### Annotations of m6A Regulators in Sarcoma

**Figure 2:**
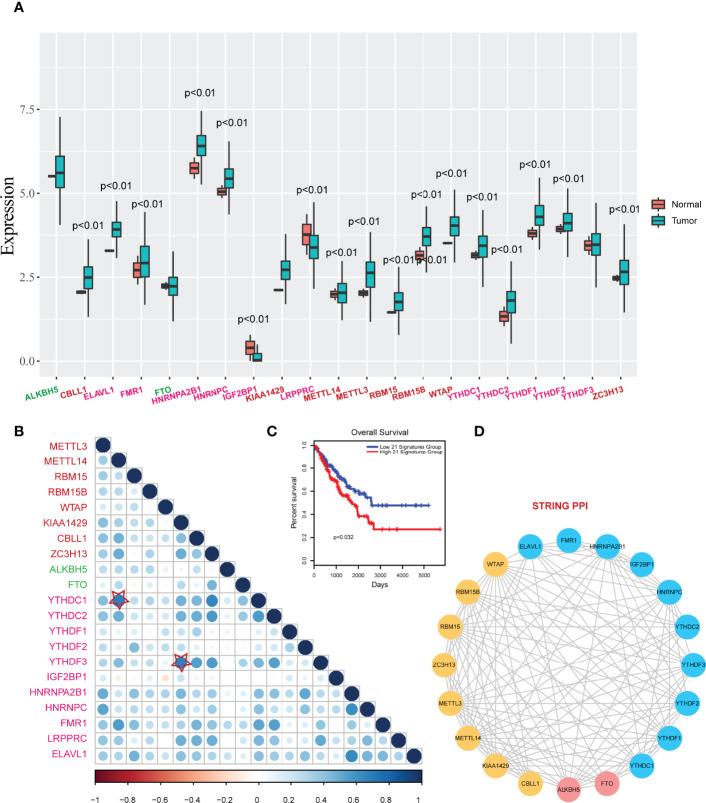
Expression changes and correlations of m6A regulators in sarcoma. **(A)** The boxplots of expression changes of m6A regulators between controls and tumors. *P*<0.01 represents the expression levels were significantly changed in tumor groups vs. control groups. **(B)** Pearson correlations of m6A regulators in sarcoma. Star-labeled nodes represent the higher crosstalks: KIAA1429- YTHDF3 and METTL14-YTHDN1. **(C)** A Kaplan-Meier survival curve of m6A regulators (risk score model) in sarcoma (P=0.032). **(D)** PPI interactions of m6A regulators in String database.

Then we viewed the RNA expression levels of m6A regulators in normal samples and tumor samples. Results showed that most of the regulators were high expressed in tumor samples, excepting IGF2P1 (**Figure 2A**). Particularly, m6A writers showed high up-regulated expression in tumor samples, such as METTL3, RBM15, RBM15B and WTAP. To investigate the relationships of m6A regulators, we performed Pearson correlation analysis for m6A regulators based on gene expression, results showed that a high correlation existed among writers, erasers, and readers (**Figure 2B**). IGF2BP1 showed low correlations to other regulators. The higher crosstalks were KIAA1429- YTHDF3 and METTL14-YTHDN1. These results implied that writers and readers were worked synergistically. Importantly, we also performed survival analysis for the 21 regulators by risk score model. Results revealed that the 21regulators model had strong prognosis effect in sarcoma (**Figure 2C**). Additionally, these writers, erasers, and readers were interacted with each other and formed a close network in String protein-protein interactions (**Figure 2D**).

**Figure 3:**
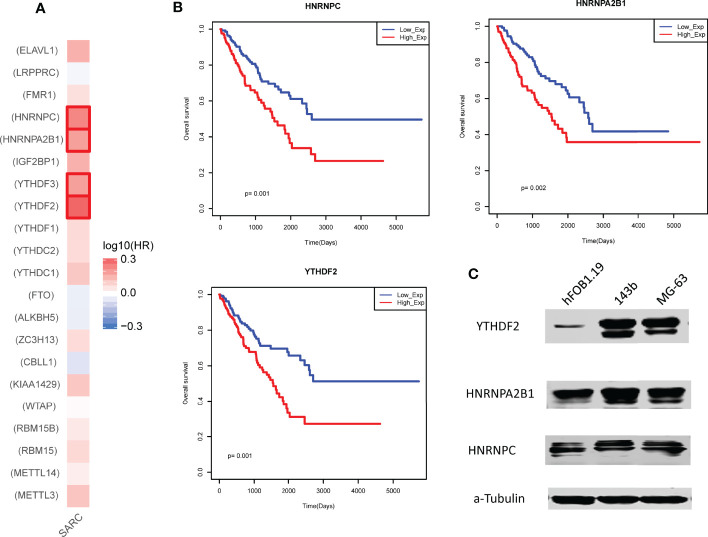
Prognostic effects of individual m6A regulator in sarcoma. **(A)** The hazard ratios of m6A regulators in sarcoma. Red marked regulators represent statistically significant risk factors (HR>1). **(B)** The Kaplan-Meier survival curve of the 3 regulators with high hazard ratios. Low_Exp group and High_Exp group were divided by mean expression. **(C)** Expression of the 3 regulators in control and model osteosarcoma cell lines. α-Tubulin was used as the reference.

We also performed single gene survival analysis for 21 m6A regulators. We mapped all these regulators into GEPIA2 database and yielded the Hazard ratios (HR) of these regulators *via* Mantel–Cox test. We found that only 4 regulators with HR >1 (**Figure 3A**). These results showed that these regulators were high risk factors in sarcoma. Overexpression of the 3 regulators (HNRNPC, HNRNPA2B1 and YTHDF2) could lead to a poor prognosis (**Figure 3B**). Furthermore, we also have tested the protein expression of the 3 regulators in control osteoblast cell lines and 2 types of osteosarcoma cell lines, results showed that all these regulators were up-regulated in sarcoma model cells (**Figure 3C**).

### Upstream Regulation Analysis of m6A Regulators

**Figure 4:**
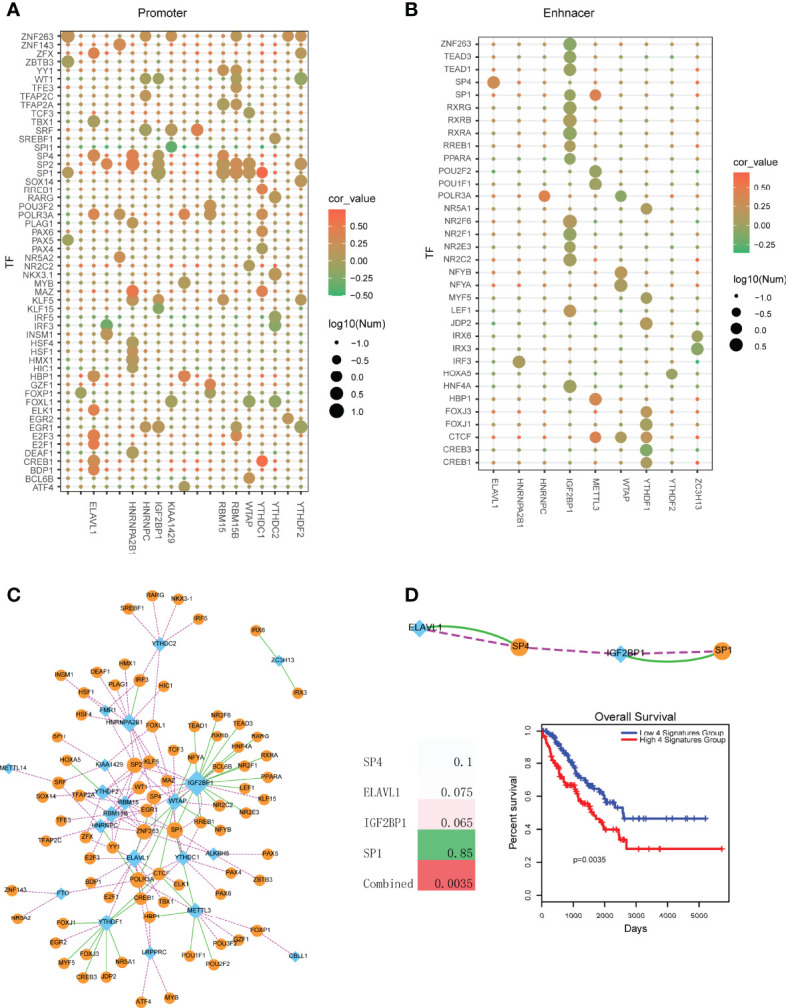
Identification of TF-m6A regulator crosstalks in sarcoma. **(A)** TF motif searching of promoter regions of m6A regulators. Node color represents the PCCs between TFs and m6A regulators. Node size represents the number of TFs that bind to the promoter regions of m6A regulators. **(B)** TF motif searching of enhancer regions of m6A regulators. Node color represents the correlation score of PCC. Node size represents the number of TFs that bind to the enhancer regions of m6A regulators. **(C)** Visualization of a TF-m6A regulator crosstalk network. Blue diamond nodes represent m6A regulators and orange circular nodes represent TFs. Green lines represent TFs binding to the enhancer regions of m6A regulators. Pink lines represent TFs binding to the promoter regions of m6A regulators. **(D)** Upper is the TF-m6A regulator crosstalks that were both regulated *via* enhancer and promoter. Lower left is the survival p-values of individual genes and combined signature in sarcoma. Lower right is the Kaplan-Meier survival curves of combined signature.

Based on the above analysis, we found that the expression levels of m6A regulators were dys-regulated between control and tumors. Thus, here we wanted to investigate the upstream regulators of these m6A regulators. Firstly, we collected all the DNA regulatory elements. Briefly, human enhancers were defined as the DNA regions of 2000bp~50000bp far from of the TSS. Promoters were defined as the regions of +/-2000bp from TSS. According to the results of motif scanning, we found that promoters were occupied more TF binding sites than enhancer (**Figures 4A, B**). YTHDC1, ELAVL1 and HNRNPA2B1 promoter regions occupied more TF binding sites that other genes. And SP family genes, such as SP1, SP2 and SP4 were the broad TFs for m6A regulators (**Figure 4A**). In enhancer perspective, results showed that binding affinity matrix was sparse (**Figure 4B**). Only the regulator of IGF2BP1 enhancer occupied more TFs. However, most of upstream TFs showed a negative correlation trend to IGF2BP1, which might explain that the expression of IGF2BP1 was opposite to other m6A regulators.

To further uncover the regulatory mechanism of TFs on m6A regulators, we then merged all the TF-m6A regulator pairs (including promoter perspective and enhancer perspective) into a network (**Figure 4C**). In this network, we found that IGF2BP1 was the biggest degree node. Some TFs, such as SP1, EGR1 and ZNF263 were the common TFs of multiple m6A regulators. Furthermore, we found that some TF-m6A regulator pairs were both regulated occurred in enhancer and promoter perspective (**Figure 4D**). The TFs of SP1 and SP4 were all demonstrated to participate in oncogenesis processes. For example, Aydemir et al. found that SP1 suppressed ADAMTS3 transcriptional activity. SP1 increased type II and III collagen expression and decreased type I collagen expression levels in Saos-2 cells. They provided the first findings for the SP1-related transcriptional regulation of ADAMTS3 and collagen genes in osteosarcoma cell lines (20). SP1 was also demonstrated to regulate lncRNA LMCD1-AS1 and lncRNA ILF3-AS1 to facilitate osteosarcoma progression (21, 22). Inhibition of SP family (SP1, SP3 and SP4) could suppress rhabdomyosarcoma cell and tumor growth *via* non-steroidal anti-inflammatory drug (NSAID) tolfenamic acid (TA) (23). Additionally, we also performed survival analysis for these common pairs. Results showed that all these single genes were not strong prognosis biomarkers (**Figure 4D**). However, combining all these genes as a single risk factor could be used as prognosis marker, which suggested the TF-m6A regulator crosstalks had the strong clinical prognostic value.

### Downstream Regulation Analysis of m6A Regulators

**Figure 5:**
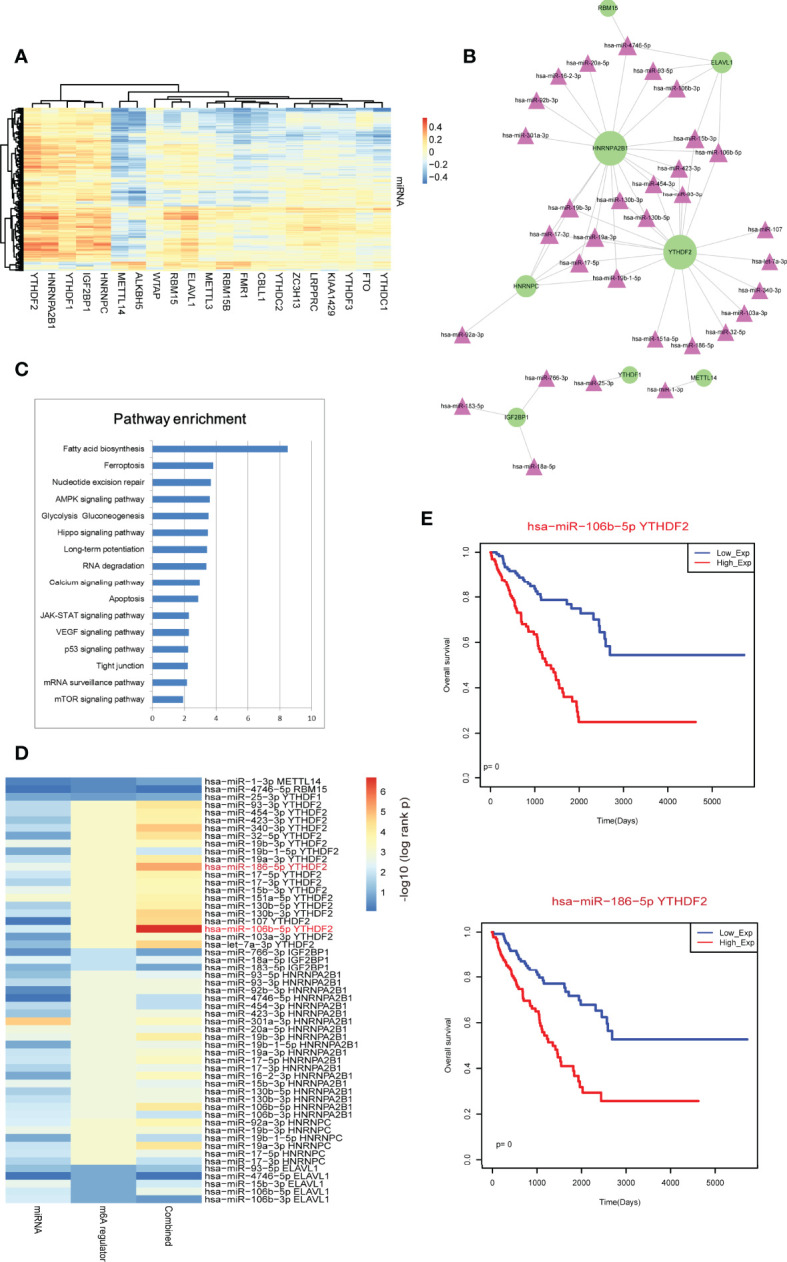
Identification of m6A regulator-miRNA pairs in sarcoma. **(A)** The Pearson correlation heatmap of miRNAs and m6A regulators. **(B)** High-correlated m6A regulator-miRNA pairs. Green circular nodes represent m6A regulators and pink triangle nodes represent miRNAs. **(C)** Pathway enrichment analysis of miRNAs in network 5B by miEAA. Pathways were ranked based on –log10 (p-value). **(D)** Survival p-values of individual genes (including single miRNA and single m6A regulator) and combined signature (risk score model) in sarcoma. **(E)** The Kaplan-Meier survival curves of strong m6A regulator-miRNA pairs.

Previous studies found that m6A regulators were the key players in miRNA processing and maturation, such as METTL3 and HNRNPA2B1 (24, 25). Thus, in this study, we investigated the potential regulatory axes between m6A regulators and miRNAs. We calculated all Pearson correlations between miRNAs and m6A regulators (**Figure 5A**). Results showed that the reader regulators, such as YTHDF2, HNRNPA2B1, YTHDF1, IGF2BP1 and HNRNPC, were high correlated with multiple miRNAs. These results were coincided with the biological function of m6A readers in RNA processing. We extracted the m6A regulator-miRNA pairs by filtering the pairs at PCC >0.4 (**Figure 5B**). We found that HNRNPA2B1 and YTHDF2 were the high-degree nodes in network. Some known pairs, such as HNRNPA2B1-miR-106b, HNRNPA2B1-miR-17 and HNRNPA2B1-miR-93 were identified from this study in sarcoma (24)[5]. We also performed miRNA function enrichment by miEAA. Results showed that multiple cancer-driven pathways were enriched, such as “Ferroptosis”, “VEGF signaling” and so on (26, 27) (**Figure 5C**). Survival analysis revealed that single miRNA could not be used as prognostic marker (**Figure 5D**). However, we then integrated m6A regulator-miRNA pair as risk score models to test the prognosis effects of these pairs, results showed that some m6A regulator-miRNA pairs had strong prognostic effects, such as YTHDF2-miR-106b-5p and YTHDF2-miR-186-5p (**Figure 5E**).

**Figure 6:**
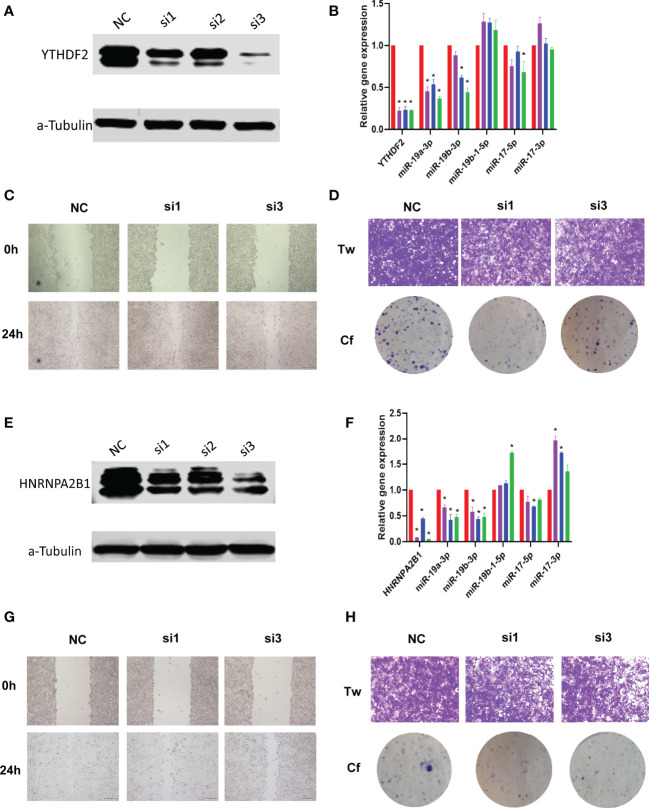
Loss of function experiments of m6A regulators in osteosarcoma cells line. **(A)** Expression of YTHDF2 in knockdown groups *via* western blot. Here we used three candidate siRNAs to target YTHDF2. **(B)** Expression of YTHDF2 and target miRNAs in knockdown groups *via* Real-time PCR. * represents *p*<0.05 vs. NC group, N=6. **(C)** Wound healing experiment results of YTHDF2 knockdown. Here we used siRNA#1 and siRNA#3. **(D)** Transwell and clone formation experiments results of YTHDF2 knockdown. Here we used siRNA#1 and siRNA#3. **(E-H)** Expression, wound healing, transwell and clone formation experiments of HNRNPA2B1 knockdown.

Importantly, to validate the biological function of m6A regulators in sarcoma, we performed loss of function experiments for two regulators, YTHDF2 and HNRNPA2B1 in osteosarcoma cell line. Results showed that the two regulators were inhibited by siRNA (**Figures 6A, E**). Inhibition of m6A regulators can affect the downstream miRNAs expression, such as miR-17 and miR-19 families (**Figures 6B, F**), which were considered as the core downstream miRNAs in **Figure 5B**. Furthermore, inhibition of m6A regulators expression can lead the tumor cell invasion and metastasis (**Figures 6C, D, G, H**).

### Immune Cell Infiltration of m6A Regulators in Sarcoma

**Figure 7:**
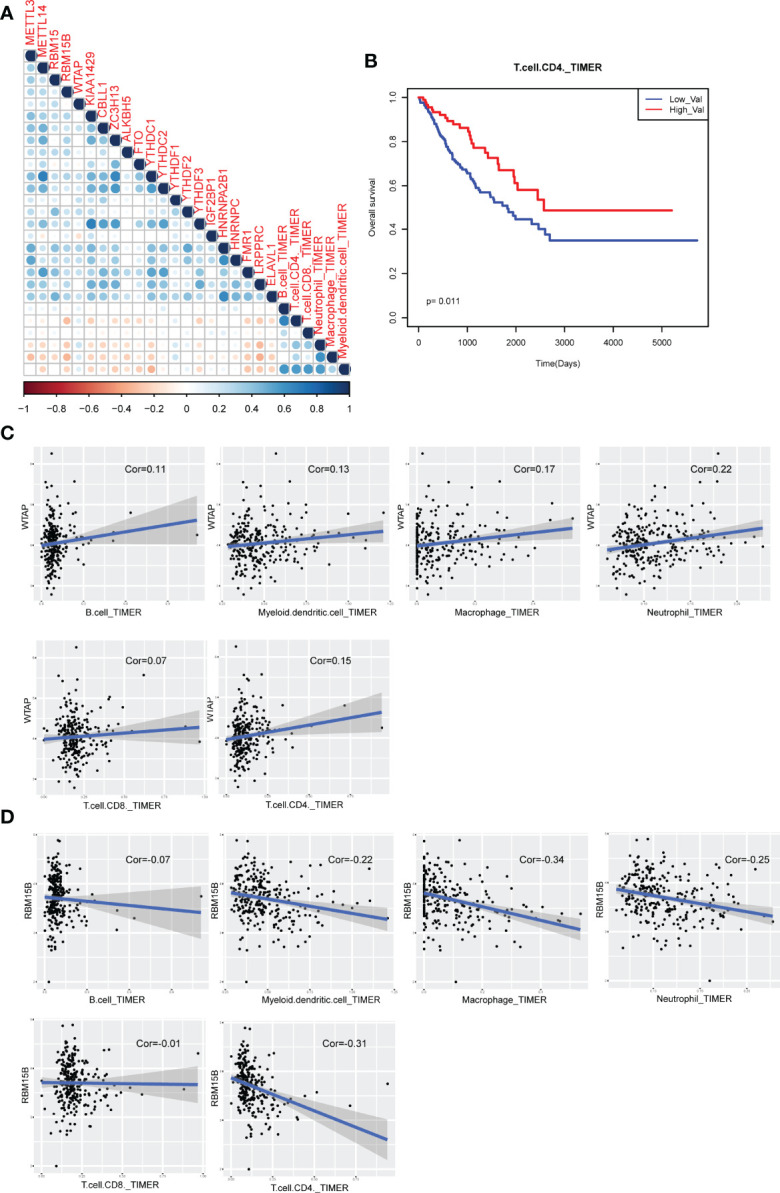
Immune cell infiltration of m6A regulators in sarcoma patients. **(A)** The visualization of correlations between m6A regulator expression and TIMER2 immune cell estimation score. **(B)** The Kaplan-Meier survival curves of between CD4 T cell-enriched patients and other patients. **(C)** Scatter plots of correlations between m6A regulator expression and TIMER2 immune cell estimation score (positive correlation). **(D)** Scatter plots of correlations between m6A regulator expression and TIMER2 immune cell estimation score (negative correlation).

Previous studies found that immune cell level determined the proliferation of cancer cells. In this study, we also investigated the association between m6A regulators and immune cell levels by calculating PCC from TIMER2 data. Results showed that most of m6A regulators were negatively correlated with immune cells (**Figure 7A**). Specifically, WTAP exhibited most positive correlation with all immune cells (**Figure 7C**). RBM15B showed most negative correlation with all immune cells (**Figure 7D**). Additionally, immune cell levels could have significant impact on clinical survival (**Figure 7B**). All these results suggested that m6A regulator might regulate cancer progression *via* controlling immune cell levels in sarcoma patients.

## Discussion

Sarcomas are rare malignant tumors that may arise from anywhere of the body, such as bone, adipose, muscle and vascular. However, the conventional pathogenesis of sarcomas has not been found. Therefore, there is an urgent need to identify novel therapeutic strategies and improve prognosis effects for sarcomas. m6A regulation is a novel proposed regulatory mechanism, which was also the most widely distributed methylation modification in eukaryotic mRNA. Growing evidence have demonstrated that m6A modification played an indispensable role in tumorigenesis. In the field of sarcoma, Zhou et al. demonstrated that knockdown of METTL3 could inhibit the proliferation and invasion of osteosarcoma by regulating ATAD2 (28). Wang et al. found that m6A played a role in the emergence and maintaining of osteosarcoma stem cells and affect the prognosis (29). Miao et al. demonstrated METTL3 promoted osteosarcoma progression by regulating the m6A level of LEF1 (30). Furthermore, m6A regulators, such as RBM15, METTL14 were also demonstrated to act as prognosis markers in multiple cancers, such as pancreatic cancer and hepatocellular carcinoma.

Here, we integrated multi-omics data including genetic alterations, gene expression and epigenomics regulation to systematically analysis the regulatory atlas of 21 m6A regulators in sarcoma. Firstly, we investigated the genetic alterations of m6A regulators and found that ~44% TCGA sarcoma patients have genetic mutations. We also investigated the basic annotation of 21 regulators, such as expression correlation and PPI interactions. Then we identified the upstream and downstream regulatory axes of m6A regulators in sarcoma based on motif analysis and mRNA-miRNA expression. These results implied that m6A regulator mediated regulatory axes could be used as prognostic biomarkers. Moreover, we also demonstrated that the expression level of m6A regulators were high related to immune cell infiltration of sarcoma patients.

Importantly, we found the expression level of m6A regulators were dys-regulated in sarcoma (**Figure 2A**). Thus, we wanted to investigate the potential mechanism of m6A regulators. One the one hand, genetic alterations could affect gene expression (**Figure 1**). On the other hand, epigenetics regulation also determined the gene expression level. We collected the distal enhancer and proximal promoters of all 21 m6A regulators and performed motif scanning to find the TF-m6A regulator crosstalks. As a result, some TFs, such as SP TF families (SP1, SP2 and SP4) were extracted as the key regulators for most of m6A regulators. We also found that promoters occupied more TFs than enhancers and m6A readers were more regulated than others, such as ELAVL1, YTHDC1 and WTAP. Additionally, some TF-m6A regulator crosstalks were both occurred in promoter and enhance perspectives, such as SP1-IGF2BP1 and SP4-ELAVL1, which also exhibited strong prognostic effects than single genes.

Recent studies found that m6A regulators were participated in miRNA maturation and processing (31, 32). In this regulatory relationship, m6A regulators showed a positive correlation with miRNAs. Thus, we calculated the PCC between miRNAs and m6A regulators. Interestingly, some known regulatory relationships were also identified in sarcoma, such as HNRNPA2B1-miR-106b, HNRNPA2B1-miR-17 and HNRNPA2B1-miR-93. m6A readers were found to positively correlate with most of miRNAs, such as HNRNPA2B1, YTHDF2, YTHDF1, IGH2BP1 and HNRNPC. Notably, m6A regulator-miRNA pairs also showed high prognostic effects. In addition, we also investigated the potential role of m6A regulators in cancer immunology. Results showed that m6A regulators might participate in cancer cell survival and cancer progression by regulating immune cell levels in sarcoma.

In summary, we systematically investigated the regulatory roles of m6A regulators in sarcoma in multi-perspectives and found the potential clinical values of m6A regulators. However, our study also exits limitations. Up to now, the massive RNA modification methylome data of sarcoma was absent. We will integrate the methylation, transcription data to analysis in the future. Furthermore, here we only used the enhancer dataset from Fantom5, which was a common enhancer of human. This is also the limitation of our current study. We will collect more enhancer datasets, such as Enhancer Atlas, to validate these results.

## Data Availability Statement

The original contributions presented in the study are included in the article/**Supplementary Material**. Further inquiries can be directed to the corresponding author.

## Author Contributions

YH designed this project, BP, DL, BC, WW, and LW processed the data and wrote the manuscript. All authors contributed to the article and approved the submitted version.

## Conflict of Interest

The authors declare that the research was conducted in the absence of any commercial or financial relationships that could be construed as a potential conflict of interest.

## Publisher’s Note

All claims expressed in this article are solely those of the authors and do not necessarily represent those of their affiliated organizations, or those of the publisher, the editors and the reviewers. Any product that may be evaluated in this article, or claim that may be made by its manufacturer, is not guaranteed or endorsed by the publisher.

## Supplementary Material

Supplementary Table 1 Raw clinical data of sarcoma cohorts in TCGA dataset.

Supplementary Table 2 siRNA sequences used in this study.

Supplementary Table 3 Primer sequences used in this study.
